# Evaluation of a modified method of extraction, purification, and characterization of lipopolysaccharide (O antigen) from *Salmonella* Typhimurium

**DOI:** 10.14202/vetworld.2020.2338-2345

**Published:** 2020-11-05

**Authors:** Heba M. Hassan, Mai A. Fadel, Mohamed A. Soliman

**Affiliations:** 1 Reference Laboratory for Veterinary Quality Control on Poultry Production, Animal Health Research Institute, Agriculture Research Center ARC, Dokki, Giza, Egypt; 2 Pharmacology and Pyrogen Unit, Department of Chemistry, Toxicology and Food Deficiency, Animal Health Research Institute, Agriculture Research Center, Dokki, Giza, Egypt

**Keywords:** extraction, high-performance liquid chromatography, interferon-γ and interleukin 1β genes expression, LPS (O antigen), purification, quantitative polymerase chain reaction, *Salmonella* Typhimurium

## Abstract

**Background and Aim:**

Lipopolysaccharide (LPS) is an integral part of the outer cell membrane complex of Gram-negative bacteria. It plays an important role in the induction and stimulation of the immune system. Various LPS purification protocols have been developed. However, analysis of their efficacy is limited by contamination during downstream applications or the public health hazard of LPS. The aim of this study was to evaluate a modified method for extracting LPS as well as assess the purity of the extracted LPS by high-performance liquid chromatography (HPLC) analysis. Further, we evaluated its immunopotentiating function by measuring the relative RNA expression levels of splenic immune-related genes such as interleukin 1β (IL-1β) and interferon-γ (IFN-γ), after intramuscular injection of increasing concentrations of the extracted LPS in specific pathogen-free (SPF) chick.

**Materials and Methods:**

Isolation, identification, and serotyping of *Salmonella* Typhimurium were performed using chicken flocks. We then performed molecular typing of *Salmonella* isolates using conventional polymerase chain reaction (PCR). A new protocol for purification of LPS from *Salmonella* isolate (*S*. Typhimurium) was conducted. HPLC analysis of the extracted LPS in the current study was compared to existing methods. An *in vivo* study was performed to evaluate the ability of LPS to induce an immune response by measuring relative IFN-γ and IL-1β gene expression after injecting increasing concentrations of the extracted LPS into SPF chicks.

**Results:**

Isolation and serotyping revealed that *Salmonella enterica* was of the serovar Typhimurium. Confirmation was conducted by molecular typing through conventional PCR. Fractionation of the LPS extract by HPLC revealed a high degree of purity comparable with standard commercial LPS. These results demonstrate the high purity of extracted LPS based on our modified method using propanol and sodium hydroxide mixture. Intramuscular injection of the extracted LPS in 22 day-old SPF chicks, compared to the negative control, revealed significant upregulation of IFN-γ and slight downregulation of IL-1β.

**Conclusion:**

The new modified method can be used for high purity LPS extraction and demonstrates effective immunopotentiating activity.

## Introduction

*Salmonella* species are members of the Enterobacteriaceae family. These organisms are Gram-negative motile flagellated bacilli and, except for *Salmonella* *gallinarum* and *Salmonella* *pullorum*, are non-motile. *Salmonella* spp. are aerobic or facultative anaerobic and their optimum growth condition is at 37°C [1,2]. Avian salmonellosis is a serious threat impacting the poultry industry. A massive increase in the spread of multiple antibiotic-resistant *Salmonella* has occurred due to the extensive use of antibiotics in human and veterinary medicine [3]. Therefore, bacterial infection control in poultry farms is a crucial step in strategic disease management. Enhancing the natural innate immunity in poultry production is an important method of disease control. Lipopolysaccharide (LPS), which is a basic component of the bacterial cell wall, has been an appropriate tool to increase the innate immunity of susceptible poultry hosts [4,5].

LPS is a major component of Gram-negative bacteria. It is a molecule consisting of lipid A, which is embedded in the outer membrane, a core oligosaccharide, and repeating O-antigen units that extend outward from the surface of the cell [6]. LPS binds to toll-like receptor 4 (TLR4), forming a pattern recognition receptor, which initiates a host innate immune response signaling pathway that results in a pro-inflammatory response involving cytokines such as interleukin 1β (IL-1β) and interferon-γ (IFN-γ) [7,8].

LPS is a molecule that is important for the virulence and pathogenesis of many bacterial species called endotoxins. Serotyping of *Salmonella* strains is differentiated based on differences in the LPS O-antigen composition [9-11]. Protocols for LPS extraction, separation, and purification have included the use of various chemical reagents such as butanol [12], ether [13], hot phenol [14], and proteinase K [15]. The hot phenol method is used to extract a high amount of LPS commercially, based on its effect on denaturing proteins and ability to lyse microorganisms. However, the hot phenol method is limited due to its biohazard nature as the steam hood should be used [16]. In this study, the propanol-sodium hydroxide method was used to extract LPS because of its speed, affordability, and safety since it does not involve use of the public health hazard, phenol. The significance of this study lies in the LPS inoculation of chickens as an effective model of inducing an inflammatory immune response to a bacterial infection without the added complications of live pathogen challenge [4].

LPS administration can activate the immune system leading to the production of high concentrations of IL-6, IL-8, IL-18, and IFN*-γ* in spleen cells [17,18]. LPS administration is associated with reduced production of cytokines, such as IL-1β, IL-6, and tumor necrosis factor [19].

The aim of this study was to evaluate the purity of the extracted LPS by high-performance liquid chromatography (HPLC) analysis as well as to evaluate the immunopotentiating function of LPS after purification by measuring the relative mRNA expression levels of splenic IL-1β and IFN-γ genes.

## Materials and Methods

### Ethical approval

All experimental bird infections were treated in accordance with the regulations for the care and husbandry concerning experimental animals and approved by the Animal Care Committee of Animal Health Research Institute.

### Samples

For *Salmonella* isolation, liver and spleen samples were aseptically collected from newly deceased or euthanized, diseased chickens of 10 flocks suffering from mortalities and diarrhea.

### Isolation and identification of *Salmonella*

We performed procedures according to ISO 6579, 2017 [20]. Pre-enrichment of the liver and spleen tissue was done in Buffered Peptone Water (Oxoid^®^, UK). The tissues were then incubated at 37°C for 16-18 h before transferring to Rappaport-Vassiliadis medium (Conda^®^,Spain). They were then incubated at 41°C for 24 h, then streaked onto XLD (LabM^®^, UK) and SS (Oxoid^®^,UK) agar plates followed by incubation at 37°C for 24 h aerobically. The typical colonies were identified by biochemical tests (urea agar, triple sugar iron, and lysine iron) (Oxoid^®^,UK).

### Serotyping of isolated *Salmonella* species

Isolates were identified in our laboratory by conventional serotyping according to the procedures of ISO 6579, 2014 [21]. We followed the White–Kauffmann–Le Minor serotyping scheme using *Salmonella* antiserum (Sifin^®^,Japan) [22].

### Confirmation of *Salmonella* Typhimurium using conventional polymerase chain reaction (PCR)

Extraction of bacterial DNA was performed using [23] oligonucleotide primers (Metabion, Germany) as follows: Forward: GGT GGC AAG GGA ATG AA and Reverse: CGC AGC GTA AAG for the target gene *S*. Typhimurium STM4495. The PCR products were separated by electrophoresis on 1% agarose gel (AppliChem, Germany, GmbH) in 1X TBE buffer at room temperature using gradients of 5 V/cm. For gel analysis, 20 μL of the PCR products were loaded in each gel slot. A 100 bp DNA ladder (Invitrogen, US) was used to determine the fragment sizes. The gel was photographed by a gel documentation system (Alpha Innotech, Biometra, Germany) and the data were analyzed through computer software.

### Extraction and purification of LPS isolated from *S. Typhimurium*

The extraction methods used were the phenol method [16] and the alcohol method [24], after corrections [25]. The third approach was based on our modified method [26]. Briefly, *S*. Typhimurium colonies were suspended in peptone water and ultracentrifuged at 10,000× *g* for 5 min. The pellets were collected, washed twice with PBS (pH 7.2), and the supernatant was discarded. *S*. Typhimurium pellets derived from the three extraction methods were the same weight (10 mg). The pellets (10 mg) were suspended in 0.5 mL of a mixture consisting of propanol:sodium hydroxide (NaOH) 1 mol/mL (5:3 v:v) with an alkaline pH of 11.4. They were kept in a 20°C water bath in a tightly closed Eppendorf tube for 2 h with gentle mixing by a magnetic stirrer. The mixture was cooled at −20°C and ultracentrifuged at 10,000× *g* for 15 min. The supernatant was collected, and the sedimented gel-like layer was extracted using 312.5 μg isopropanol and ultracentrifuged at 10,000× *g* for 5 min. The precipitate was discarded and the supernatant was added to the previous supernatant and diluted with an equal volume of distilled water. The sample was centrifuged at 2000 rpm for 5 min. The resulting supernatant was collected and injected on HPLC.

### Characterizing LPS isolated from *S. Typhimurium* through HPLC

The Agilent Series 1200 quaternary gradient pump, Series 1200 autosampler, Series 1200 UV detector, and HPLC 2D ChemStation software (Hewlett-Packard, Les Ulis, France) were used in this study. Chromatographic separation was performed on a reversed-phase column (Dionex Acclaim TM 120, C18 [150×4.6 mm, 5 μm]) with a mixture of water and methanol (65:35) as the mobile phase. Extra pure *S*. Typhimurium LPS (Sigma, Saint Louis, USA) was used as the standard [27].

### Standard preparation and chromatographic separation

**Figure-1:**
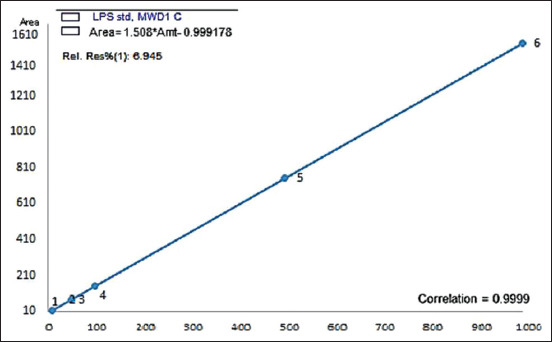
Linearity of lipopolysaccharide standard in deionized water.

A stock standard solution was prepared by dissolving 1 mg of lyophilized LPS in deionized water to prepare the following concentrations 0.005, 0.01, 0.05, 0.1, 0.5, and 1 μg/mL within the correlation coefficient (r^2^=0.9999), as shown in Figure-1. The flow rate was 1 mL/min with a UV detector at a wavelength of 210 nm. The analyte was injected on HPLC with an injection volume of 10 μL and an ambient column temperature. The output of the recovery percent test was used to determine the amount of LPS yielded from the three extraction methods being evaluated. Standard addition is an effective way to evaluate accuracy by adding 10 ng/g of pure LPS standard to the extracted, known mean concentrations (45.5, 48.42, and 50.12 ng/g) for the three methods. Standard addition can also be used to assess whether a sample has a matrix effect. If the sample does have a matrix effect, the standard addition procedure provides a more accurate measurement of the concentration of the analyte in the sample than the use of a standard curve [28]. The accuracy of the modified method [26] was assessed by analyzing six replicates of LPS standard calibration curves during a single day. Recovery studies were performed by calculating the recovery and relative standard deviation (RSD%). The recovery percent acceptance criteria should range between 98% and 102% and the RSD% should be <1% [29].

### *In vivo* analysis of LPS administration

The chickens were divided into five groups (four experimental and one control group) of five chickens each at 22 days old. Different concentrations (50, 100, 15, and 200 μg/mL) of LPS were inoculated intramuscularly and the control group was injected with saline. Each group was housed in separate controlled biosafety isolators at the Reference Laboratory for Veterinary Quality Control on Poultry Production (RLQP) experiment animal house. Birds were fed antibiotic-free rations and supplied with water *ad libitum*. A day before LPS injection, samples were collected and tested for *Salmonella*. After injection, the chicks were kept for 12 h and then euthanized to prepare for spleen collection. Spleens were stored in RNA later solution.

### Quantitative measurement of IL-1β and IFN-γ gene expression

#### RNA extraction

RNA was extracted from spleen samples in triplicate using the Biamp RNeasy Mini kit (Qiagen, Germany, GmbH) when 30 mg of the spleen tissue sample was added to 600 μL lysis buffer (RLT) containing 10 μL β-mercaptoethanol per mL. To homogenize the samples, tubes were placed into the adaptor sets, which were fixed into the clamps of the Qiagen TissueLyser. Disruption was performed for 2 min at high speed (30 Hz), with a shaking step. One volume of 70% ethanol was added to the cleared lysate, and purification of total RNA from animal tissues was performed according to the manufacturer’s protocol of the QIAamp RNeasy Mini kit (Qiagen, Germany, GmbH).

#### Oligonucleotides

The primers and probes used were supplied from Metabion (Germany) and are shown in Table-1 [30,31].

**Table-1:** Primers and probes sequences, target genes, and cycling conditions for TaqMan RT-PCR.

Target gene	Primers and probes sequences (5’-3’)	Reverse transcription	Primary denaturation	Amplification (40 cycles)		References

				Secondary denaturation	Annealing and extension	
*28S* *rRNA*	Forward: GGCGAAGC CAGAGGAAACT	50°C 30 min	94°C 15 min	94°C 15 s	60°C 1 min	[30]
	Reverse: GACGACC GATTTGCACGTC					
	Probe: (FAM) AGGACCGCTA CGGACCTCCACCA (TAMRA)					
*IFN- γ*	Forward: GTGAAGAAGGT GAAAGATATCATGGA					
	Reverse: GCTTTGCGCTG GATTCTCA					
	Probe: (FAM) GGCCAAGC TCCCGATGAACGA (TAMRA)					
*IL1β*	Forward: GCTCTACATGT CGTGTGTGATGAG					[31]
	Reverse: TGTCGATGTC CCGCATGA					
	Probe:(FAM) CCACACTGCAGCTGGA GGAAGCC (TAMRA)					

IFN-γ=Interferon-γ, IL-1β=Interleukin 1β, RT-PCR=Reverse transcription polymerase chain reaction

### TaqMan RT-PCR

PCR amplification was performed in a final volume of 25 μL containing 3 μL of RNA template, 12.5 μL of 2× QuantiTect Probe RT-PCR Master Mix, 8.125 0.125 μL of each probe (30 pmol), and 0.25 μL of QuantiTect RT Mix. The reaction was performed in a Stratagene MX3005P real-time PCR machine.

### Analysis of qRT-PCR results

Amplification curves and threshold cycle (Ct) values were determined using the Stratagene MX3005P software. To estimate the quantitative RNA variation of IFN-γ and IL-1β gene expression for each sample, the mean Ct values (of the triplicates) were calculated within each sample for each gene. For the two test genes, expression was normalized to delta Ct (ΔCt) using the geometric mean. Mean ΔCt values were determined for each group compared to the expression of 18S rRNA as the endogenous control. Relative expression levels (fold change) were calculated for each comparison between groups using the delta delta Ct (2^−ΔΔ*C*t^) method, as previously published [32]. Fold changes were log transformed (log_2_FC) and used to determine pairwise correlations with significance levels of p≤0.05.

## Results

### Isolation, identification, and serogrouping

Seven *Salmonella* isolates were detected from the examined samples and all were identified serologically as *S*. Typhimurium 1,4,5,12:i:1,2.

### Confirmation of S. Typhimurium using conventional PCR

**Figure-2:**
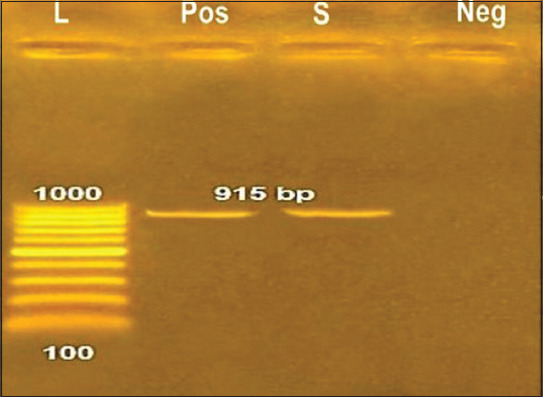
Amplified *Salmonella* extract lane (S) at the molecular weight as positive control Salmonella reference strain (pos) in 915 bp using conventional polymerase chain reaction.

In the current study, the *STM-4495* gene was detected in the isolates of *S*. Typhimurium using corresponding specific primers that produced amplicons with a molecular weight of 915 bp (Figure-2).

### Characterization of LPS isolated from S. Typhimurium using HPLC

**Figure-3:**
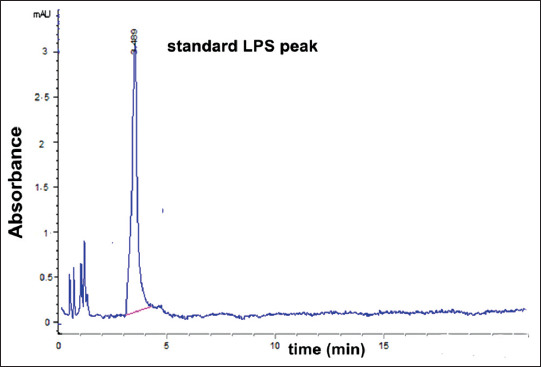
High-performance liquid chromatography chromatogram of standard lipopolysaccharide for *Salmonella* Typhimurium (50 µg/kg) concentration.

**Figure-4:**
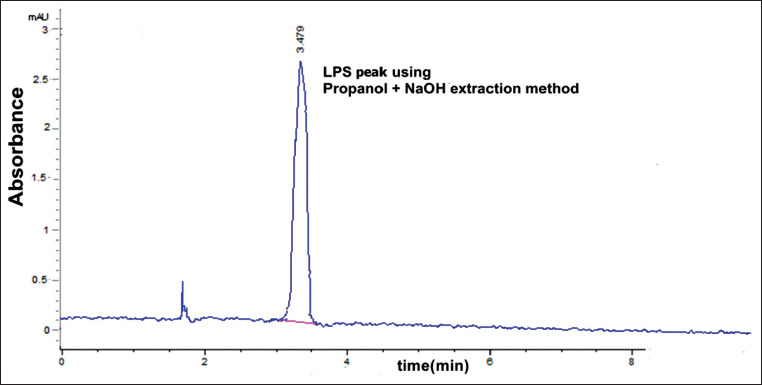
High-performance liquid chromatography chromatogram of extracted Lipopolysaccharide from *Salmonella* Typhimurium using propanol and NaOH method (50 µg/kg) concentration.

**Figure-5:**
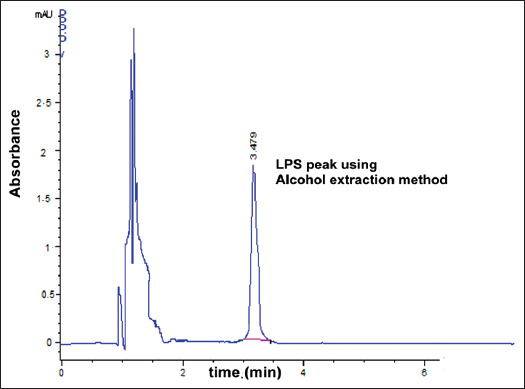
High-performance liquid chromatography chromatogram of extracted Lipopolysaccharide from *Salmonella* Typhimurium using alcohol extraction method (50 µg/kg) concentration.

**Figure-6:**
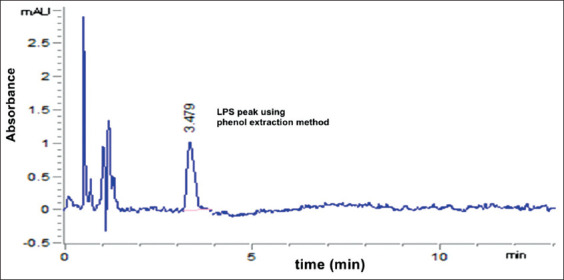
High-performance liquid chromatography chromatogram of extracted Lipopolysaccharide from *Salmonella* Typhimurium by phenol extraction method (50 µg/kg) concentration.

The best peak resolution was seen at a retention time of 3.479 min. The observed accuracy of the propanol-sodium hydroxide method was 100.16±0.6. The purity of the extracted LPS was assessed by HPLC. The extracted LPS from *S*. Typhimurium was analyzed and compared to that of the extra pure commercial standard, as shown in Figure-3. Using the modified propanol-sodium hydroxide method, the HPLC chromatogram of the extracted LPS from *S*. Typhimurium is shown overlaid with that of the LPS standard, indicating the high purity of the product (Figure-4). Furthermore, this chromatogram showed a single major sharp band suggesting very low content of impurities. LPS generated by the alcohol and phenol extraction methods demonstrated differences in the area compared to the LPS standard, which explains the lower recovery percent than the developed propanol-sodium hydroxide method (Figures-5 and 6). This difference may be due the matrix effect of the two other methods. This was determined through standard addition by known concentration (Table-2) [16,24,26].

**Table-2:** Recovery studies for intraday precision of the three comparable methods and standard addition with known concentration (10 ng/g).

Methods	Extracted LPS resulted conc. (ng/g) with standard addition	Recovery %	Recovery % mean±SD	Relative standard deviation RSD%
Phenol method [16]	54.81108	91.35179	91.99±1.137393	1.209106
	54.52369	90.87282		
	56.22793	93.71321		
	54.88754	91.47924		
	55.51651	92.52752		
	55.86228	93.10379		
Alcohol method [24]	58.62296	97.70493	98.0±0.574954	0.586431
	59.02873	98.38122		
	58.55816	97.59694		
	59.29013	98.81689		
	58.49397	97.48996		
	58.96054	98.26756		
Propanol and NaOH method [26]	60.34428	100.5738	100.2±0.267508	0.288729
	59.97293	99.95488		
	60.27134	100.4522		
	60.08122	100.1354		
	60.02731	100.0455		
	59.90065	99.83441		

LPS=Lipopolysaccharide, RSD=Relative standard deviation

Our results show that the best method for LPS extraction of the three methods evaluated was our method developed using a mixture of alcohol and sodium hydroxide followed by the alcohol method, with the least effective method being the phenol method. Each gram of bacterial pellets yielded a mean concentration of 6.1 μg/g pure commercial LPS in our developed propanol-sodium hydroxide method. However, the yielded mean concentrations of LPS extracted by the phenol and alcohol methods were 3.001 and 4.85 μg/g, respectively, for the same weight of bacterial pellets. The RSD% was ≤1 % to our modified method but Ismail *et al*. [24], exceeded this RSD% with the phenol method.

### Quantitative measurement of IL-1β and IFN-γ gene expression

**Figure-7:**
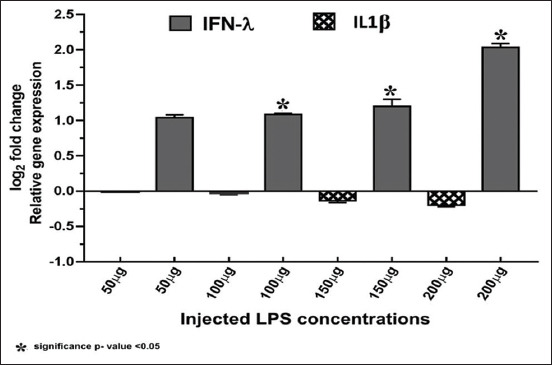
Relative quantification (RQ) gene expression of interferon-γ (IFN-γ) and interleukin 1β (IL-1β) (log_2_ fold change) showed upregulation of IFN-γ expressed gene in correlation with ascending injected concentrations of Lipopolysaccharide (50, 100, 150, and 200 µg) and downregulation of IL-1β gene. The RQ of each gene, expressed as log_2_ fold change, was obtained using the (2-^ΔΔCt^) method and by normalizing the mRNA expression to 18S rRNA (*p≤value).

Table-3 shows significant upregulation of IFN-γ gene expression with increasing LPS concentration. However, IL-1β gene expression showed slight downregulation with increasing concentration of LPS (Figure-7).

**Table-3:** IL-1β and IFN-γ splenic gene expression correlated with ascending injecting concentration of LPS. The relative level of each gene, expressed as fold change, was obtained using the (2^-ΔΔ*C*t^) method.

IFN-γ fold change	IL-1β fold change	LPS conc. µg/mL
50	0.99	1.07
100	0.95	1.09
150	0.84	1.30
200	0.73	2.12

LPS=Lipopolysaccharide, IFN-γ=Interferon-γ, IL-1β=Interleukin 1β

## Discussion

*Salmonella* *enterica* serovars are important model organisms that induce bacterial pathogenesis and increase innate immunity [8]. As a consequence of host infection with *S*. Typhimurium, its outer membrane structure (LPS) can trigger an immune cytokine storm through the pattern recognition receptor, TLR4 [8]. The previous studies have shown that pure LPS can induce a strong reaction alone or through phagocytic cells from the macrophage-monocyte group, which are sensitive to *in vivo* injection of LPS and can produce inflammatory molecules such as IFN-γ and IL-1β [33,34]. A number of methods for extraction, separation, and purification of LPS exist. However, impurities, low yield, or public health concerns are considered as drawbacks for these methods.

In the present study, we evaluated a developed extraction method for LPS and its detection using HPLC and compared the results to traditional LPS extraction methods, the phenol method [16], and the alcohol method [24]. We used a propanol and sodium hydroxide mixture to yield LPS recovery on HPLC that was significantly greater than the other two methods tested (hot phenol and alcohol methods). The retention factor of the modified method (propanol-sodium hydroxide; Figure-4) was equal to the LPS standard (Figure-3) and greater than the other two methods (Figures-5 and 6). As shown in Table-2, the developed propanol-sodium hydroxide extraction method yielded a mean concentration of 6.1 μg/g of bacterial pellets. However, the other two methods yielded 4.85 and 3.01 μg/g mean concentrations of LPS for the same weight of bacterial pellets. Therefore, the current study provides a comparison of three LPS extraction methods proving high yielded mean concentration of LPS compared to a previous study by Rezania *et al*. [35], which was based only on the phenol method. The main argument against the phenol method was provided by Seite *et al*. [36] who mentioned that the deproteinizing action of the hot 45% aqueous phenol on whole cells or isolated and purified endotoxin is caused by the cleavage of a phenol sensitive linkage within the lipid moiety. As a result of this degradation, both the LPS and simple protein fragments retained a part of the lipid moiety. Although not proceeding at the same fast rate as the cleavage of the lipid moiety, this phenol treatment also caused partial hydrolysis of the O-specific side chain and ester-bound fatty acids. This can be explained by the presence of degradation products, which is a main cause of the impurity of the LPS extraction. In this study, we also evaluated LPS extraction through the alcohol method [24], in which the precipitate and concentrate LPS fractions were described by Perdomo and Montero [37]. Therefore, sodium hydroxide was chosen as the best chemical to produce LPS with free DNA content [26,38]. Here, we show that this modified method using sodium hydroxide produced the best quality and the highest purity LPS compared to the phenol or the alcohol method.

The *in vivo* experiment was performed to demonstrate the effectiveness of the immunopotentiating impact of the extracted LPS in young SPF chicks. We show elevated splenic RNA expression levels of IFN-γ with reduced RNA expression of IL-1β. This immune response in chicks was observed at 12 h post-injection in accordance with the previous studies [39,40]. These findings accomplish the aim to produce a new protocol for extraction and purification of LPS with the highest purity and minimum contamination. In addition, we demonstrate that this protocol preserved the biological effectiveness of LPS without any harmful effects or functional impact to the generated LPS extract.

## Conclusion

The protocol presented here can be used to isolate LPS with high purity and functional activity from different strains of smooth Gram-negative bacteria, which have structurally different LPS. The expression of INF-γ and IL-1β genes following LPS stimulation clearly indicates its immunostimulating effect in young chicks. Future studies on the potential implications of LPS in improving natural immunity and tolerance to disease infection in the poultry field are, therefore, warranted.

## Authors’ Contributions

HMH and MAF designed the study. MAS designed the *in-vivo* experiment. HMH performed the bacterial isolation and typing. MAF performed the extraction, purification and HPLC analysis of LPS. MAS performed the molecular typing and the quantitative genes expression. HMH, MAF and MAS analyzed the data, drafted the manuscript, revised and finalized the manuscript for submission. All authors read and approved the final manuscript.
